# QuickStats

**Published:** 2013-07-12

**Authors:** Yelena Gorina

**Figure:**
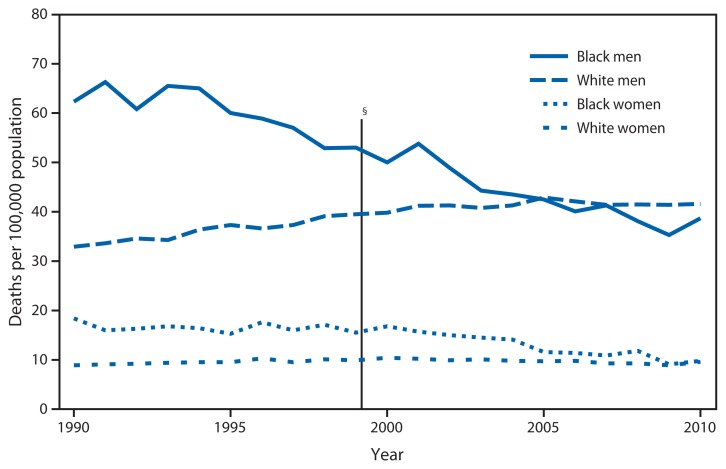
Age-Adjusted Death Rates^*^ from Esophageal Cancer^†^ for Persons Aged ≥65 Years, by Race and Sex — National Vital Statistics System, United States, 1990–2010 ^*^ Per 100,000 population. Rates have been revised by using populations enumerated as of April 1, for 2000 and 2010, and intercensal estimates as of July 1 for all other years. Therefore, the rates might differ from those published previously. ^†^ Deaths from esophageal cancer include those coded as C15 in the *International Classification of Diseases, 10th Revision* (ICD-10) and as 150 in the *International Classification of Diseases, Ninth Revision* (ICD-9). ^§^ In 1999, ICD-10 replaced the ICD-9. Little change was observed in the classification of esophageal cancer deaths from ICD-9 to ICD-10.

During 1990–2010, the age-adjusted esophageal cancer death rate decreased 38% for black men and 47% for black women aged ≥65 years. For white men in this age group, the rates increased 26% during 1990–2002 and stabilized during the rest of the decade; for white women the rates stayed nearly the same. In 2010, esophageal cancer death rates were nearly 40 per 100,000 population for white and black men aged ≥65 years and nearly 10 per 100,000 population for white and black women in the same age group.

**Sources:** CDC. National Vital Statistics System. Available at http://www.cdc.gov/nchs/data_access/vitalstatsonline.htm.

CDC. Health Data Interactive. Available at http://www.cdc.gov/nchs/hdi.htm.

CDC. CDC WONDER. Available at http://wonder.cdc.gov.

